# Supplementary material for: Using Species Distribution Models (SDMs) to Estimate the Suitability of European Mediterranean Non-Native Area for the Establishment of Toumeyella Parvicornis (Hemiptera: Coccidae)
Source: Insects. 2023 Jan 3;14(1):46. doi: 10.3390/insects14010046 (PMC9862868; doi:10.3390/insects14010046)
Supplement: Supplementary file 1 [file insects-14-00046-s001.zip › insects-2106165-Supplementary S1.pdf]

Supplementary material S1. List of records used to estimate the suitability of European Mediterranean non-native area for *Toumeyella parvicornis* using Species Distribution Models (SDMs).

Country	Region	Municipality	Latitude	Longitude	Source
Italy	Lazio	Nettuno	41.512	12.664	Visual inspection
Italy	Lazio	Anzio	41.506	12.621	Visual inspection
Italy	Lazio	Anzio	41.509	12.626	Visual inspection
Italy	Lazio	Lido dei Pini	41.527	12.571	Visual inspection
Italy	Lazio	Marina di Ardea	41.550	12.549	Visual inspection
Italy	Lazio	Pomezia	41.646	12.484	Visual inspection
Italy	Lazio	Velletri	41.651	12.785	Visual inspection
Italy	Lazio	Castel Porziano	41.684	12.440	Visual inspection
Italy	Lazio	Castel Porziano	41.766	12.398	Visual inspection
Italy	Lazio	Castel Fusano	41.738	12.327	Visual inspection
Italy	Lazio	Ostia Antica	41.758	12.294	Visual inspection
Italy	Lazio	Ostia Antica	41.791	12.326	Visual inspection
Italy	Lazio	Ciampino	41.795	12.584	Visual inspection
Italy	Lazio	Tor Bella Monaca	41.870	12.639	Visual inspection
Italy	Lazio	Tor Bella Monaca	41.867	12.661	Visual inspection
Italy	Lazio	Lunghezza	41.912	12.666	Visual inspection
Italy	Lazio	Focene	41.796	12.228	Visual inspection
Italy	Lazio	Fregene	41.856	12.202	Visual inspection
Italy	Lazio	Castel di Guido	41.901	12.279	Visual inspection
Italy	Lazio	Pantan Monastero	41.925	12.353	Visual inspection
Italy	Lazio	Cerveteri	41.980	12.141	Visual inspection
Italy	Lazio	Marcigliana	42.032	12.549	Visual inspection
Italy	Lazio	Labaro	41.995	12.485	Visual inspection
Italy	Lazio	Monte Sacro (Roma)	41.934	12.532	Visual inspection

Italy	Lazio	Cinecittà (Roma)	41.854	12.557	Visual inspection
Italy	Lazio	Tor Marancia (Roma)	41.851	12.498	Visual inspection
Italy	Lazio	La Pisana (Roma)	41.853	12.381	Visual inspection
Italy	Lazio	Pineta Sacchetti (Roma)	41.907	12.426	Visual inspection
Italy	Lazio	Borgo Piave	41.478	12.852	Visual inspection
Italy	Lazio	Borgo Piave	41.471	12.879	Visual inspection
Italy	Lazio	Campo di Carne	41.539	12.635	Visual inspection
Italy	Lazio	Acciarella	41.444	12.747	Visual inspection
Italy	Lazio	Cisterna di Latina	41.558	12.818	Visual inspection
Italy	Lazio	Formia	41.250	13.589	Visual inspection
Italy	Lazio	Cassino	41.484	13.822	Visual inspection
Italy	Lazio	Cassino	41.499	13.838	Visual inspection
Italy	Campania	Acerra	40.934	14.366	Servizio Fitosanitario Regione Campania
Italy	Campania	Arzano	40.917	14.274	Servizio Fitosanitario Regione Campania
Italy	Campania	Caivano	40.966	14.303	Servizio Fitosanitario Regione Campania
Italy	Campania	Cercol	40.853	14.353	Servizio Fitosanitario Regione Campania
Italy	Campania	Ercolano	40.820	14.378	Servizio Fitosanitario Regione Campania
Italy	Campania	Giugliano in Campania	40.929	14.039	Servizio Fitosanitario Regione Campania
Italy	Campania	Napoli	40.826	14.188	Servizio Fitosanitario Regione Campania
Italy	Campania	Napoli	40.837	14.312	Servizio Fitosanitario Regione Campania
Italy	Campania	Napoli	40.837	14.314	Servizio Fitosanitario Regione Campania
Italy	Campania	Napoli	40.814	14.199	Servizio Fitosanitario Regione Campania
Italy	Campania	Napoli	40.820	14.203	Servizio Fitosanitario Regione Campania
Italy	Campania	Portici	40.812	14.344	Servizio Fitosanitario Regione Campania
Italy	Campania	San Sebastiano Vesuvio	40.837	14.369	Servizio Fitosanitario Regione Campania
Italy	Campania	Torre del Greco	40.798	14.383	Servizio Fitosanitario Regione Campania
Italy	Abruzzo	Pescara	42.457	14.233	EPPO database
France	Côte d'Azur	Saint-Tropez	43.268	6.655	EPPO database
