# Supplementary material for: Using Species Distribution Models (SDMs) to Estimate the Suitability of European Mediterranean Non-Native Area for the Establishment of Toumeyella Parvicornis (Hemiptera: Coccidae)
Source: Insects. 2023 Jan 3;14(1):46. doi: 10.3390/insects14010046 (PMC9862868; doi:10.3390/insects14010046)
Supplement: Supplementary file 1 [file insects-14-00046-s001.zip › insects-2106165-Supplementary S2.pdf]

Supplementary materials S2. Results of Pearson's correlation analysis among bioclimatic variables at a spatial resolution of 30 arc-sec (~1 km^2).

	**BIO01**	**BIO02**	**BIO03**	**BIO04**	**BIO05**	**BIO06**	**BIO07**	**BIO08**	**BIO09**	**BIO10**	**BIO11**	**BIO12**	**BIO13**	**BIO14**	**BIO15**	**BIO16**	**BIO17**	**BIO18**	**BIO19**
**BIO01**	1.000	0.319	0.449	-0.055	0.955	0.932	0.106	0.577	0.695	0.982	0.962	-0.405	-0.236	-0.545	0.405	-0.275	-0.491	-0.645	0.009
**BIO02**	0.319	1.000	0.738	0.533	0.542	0.030	0.821	0.474	-0.050	0.419	0.157	0.031	-0.008	0.157	-0.236	-0.046	0.187	0.137	-0.203
**BIO03**	0.449	0.738	1.000	-0.158	0.496	0.374	0.224	0.296	0.344	0.416	0.466	-0.126	-0.033	-0.244	0.275	-0.036	-0.210	-0.206	-0.018
**BIO04**	-0.055	0.533	-0.158	1.000	0.203	-0.386	0.912	0.393	-0.520	0.135	-0.322	0.189	0.022	0.505	-0.634	-0.026	0.502	0.460	-0.284
**BIO05**	0.955	0.542	0.496	0.203	1.000	0.796	0.386	0.637	0.563	0.987	0.852	-0.333	-0.213	-0.382	0.209	-0.264	-0.325	-0.507	-0.057
**BIO06**	0.932	0.030	0.374	-0.386	0.796	1.000	-0.251	0.364	0.827	0.853	0.990	-0.438	-0.227	-0.675	0.584	-0.245	-0.624	-0.770	0.131
**BIO07**	0.106	0.821	0.224	0.912	0.386	-0.251	1.000	0.464	-0.360	0.279	-0.147	0.135	0.005	0.417	-0.556	-0.049	0.431	0.362	-0.291
**BIO08**	0.577	0.474	0.296	0.393	0.637	0.364	0.464	1.000	-0.017	0.641	0.431	-0.340	-0.307	-0.253	0.093	-0.329	-0.262	-0.060	-0.480
**BIO09**	0.695	-0.050	0.344	-0.520	0.563	0.827	-0.360	-0.017	1.000	0.594	0.805	-0.288	-0.066	-0.584	0.521	-0.098	-0.489	-0.766	0.337
**BIO10**	0.982	0.419	0.416	0.135	0.987	0.853	0.279	0.641	0.594	1.000	0.894	-0.365	-0.230	-0.441	0.277	-0.278	-0.388	-0.553	-0.043
**BIO11**	0.962	0.157	0.466	-0.322	0.852	0.990	-0.147	0.431	0.805	0.894	1.000	-0.439	-0.237	-0.652	0.550	-0.260	-0.601	-0.742	0.086
**BIO12**	-0.405	0.031	-0.126	0.189	-0.333	-0.438	0.135	-0.340	-0.288	-0.365	-0.439	1.000	0.927	0.767	-0.306	0.936	0.834	0.724	0.695
**BIO13**	-0.236	-0.008	-0.033	0.022	-0.213	-0.227	0.005	-0.307	-0.066	-0.230	-0.237	0.927	1.000	0.526	-0.013	0.982	0.628	0.521	0.790
**BIO14**	-0.545	0.157	-0.244	0.505	-0.382	-0.675	0.417	-0.253	-0.584	-0.441	-0.652	0.767	0.526	1.000	-0.785	0.523	0.971	0.818	0.235
**BIO15**	0.405	-0.236	0.275	-0.634	0.209	0.584	-0.556	0.093	0.521	0.277	0.550	-0.306	-0.013	-0.785	1.000	0.012	-0.742	-0.532	0.177
**BIO16**	-0.275	-0.046	-0.036	-0.026	-0.264	-0.245	-0.049	-0.329	-0.098	-0.278	-0.260	0.936	0.982	0.523	0.012	1.000	0.609	0.550	0.804
**BIO17**	-0.491	0.187	-0.210	0.502	-0.325	-0.624	0.431	-0.262	-0.489	-0.388	-0.601	0.834	0.628	0.971	-0.742	0.609	1.000	0.788	0.337
**BIO18**	-0.645	0.137	-0.206	0.460	-0.507	-0.770	0.362	-0.060	-0.766	-0.553	-0.742	0.724	0.521	0.818	-0.532	0.550	0.788	1.000	0.046
**BIO19**	0.009	-0.203	-0.018	-0.284	-0.057	0.131	-0.291	-0.480	0.337	-0.043	0.086	0.695	0.790	0.235	0.177	0.804	0.337	0.046	1.000
